# Astrocyte-Specific Disruption of SynCAM1 Signaling Results in ADHD-Like Behavioral Manifestations

**DOI:** 10.1371/journal.pone.0036424

**Published:** 2012-04-30

**Authors:** Ursula S. Sandau, Zefora Alderman, Gabriel Corfas, Sergio R. Ojeda, Jacob Raber

**Affiliations:** 1 Division of Neuroscience, Oregon National Primate Research Center, Oregon Health & Science University, Beaverton, Oregon, United States of America; 2 Departments of Behavioral Neurosciences and Neurology, Oregon Health & Science University, Portland, Oregon, United States of America; 3 F. M. Kirby Neurobiology Program, Harvard Medical School, Children's Hospital, Boston, Massachusetts, United States of America; Zhejiang University School of Medicine, China

## Abstract

SynCAM1 is an adhesion molecule involved in synaptic differentiation and organization. SynCAM1 is also expressed in astroglial cells where it mediates astrocyte-to astrocyte and glial-neuronal adhesive communication. In astrocytes, SynCAM1 is functionally linked to erbB4 receptors, which are involved in the control of both neuronal/glial development and mature neuronal and glial function. Here we report that mice carrying a dominant-negative form of SynCAM1 specifically targeted to astrocytes (termed GFAP-DNSynCAM1 mice) exhibit disrupted diurnal locomotor activity with enhanced and more frequent episodes of activity than control littermates during the day (when the animals are normally sleeping) accompanied by shorter periods of rest. GFAP-DNSynCAM1 mice also display high levels of basal activity in the dark period (the rodent's awake/active time) that are attenuated by the psychostimulant D,L-amphetamine, and reduced anxiety levels in response to both avoidable and unavoidable provoking stimuli. These results indicate that disruption of SynCAM1-dependent astroglial function results in behavioral abnormalities similar to those described in animals model of attention-deficit hyperactive disorder (ADHD), and suggest a hitherto unappreciated contribution of glial cells to the pathophysiology of this disorder.

## Introduction

SynCAM1 is a member of the immunoglobulin (Ig) superfamily, a large group of proteins involved in cell surface recognition [1], [2]. In vertebrates, four SynCAM genes, encoding highly conserved intracellular and extracellular domains have been described [3]. One of these genes encodes SynCAM1, a protein originally described as “tumor-suppressor in lung cancer −1” (TSLC1) [4], [5], and that is also known as nectin-like protein 3 (Necl2), or Igsuperfamily4 (IGSF4). SynCAM1 plays an important role in central nervous system development because it promotes synaptic assembly [6], induces functional differentiation of presynaptic terminals [7], enhances excitatory synaptic transmission [7], [8], mediates the organization of adhesive contacts between neuronal growth cones and neurites [9], and maintains newly formed excitatory synapses [10].

Although SynCAM1 is a major synaptic adhesive protein, we recently found that SynCAM1 is also produced in astrocytes where it plays a major role in facilitating astrocyte-to-astrocyte and astrocyte-to-neuron adhesive communication [11]. We also showed that SynCAM1 adhesive behavior is functionally coupled to the tyrosine kinase receptor erbB4, a cell membrane protein that recognizes neuregulin-1 as a ligand [12], [13] and that is co-expressed with SynCAM1 in astrocytes [11], [14]. Ligand-dependent activation of astrocytic erbB4 receptors results in a rapid, but transient, increase in SynCAM1 adhesive behavior. Conversely, disruption of astrocytic erbB4 receptor function leads to loss of SynCAM1-mediated adhesiveness [11]. Because of our interest in the neuroendocrine control of reproductive development, we wanted to determine if astrocytic SynCAM1-dependent signaling is required for normal female reproductive function. Therefore, we generated transgenic mice that express – in an astrocyte-specific manner – a dominant-negative form of SynCAM1 (GFAP-DNSynCAM1) lacking the intracellular domain [14]. We observed that female mice carrying this transgene had a delayed onset of puberty, disrupted estrous cyclicity and reduced fecundity. These deficits were associated with a reduced capacity of hypothalamic astrocytes to respond to erbB4-mediated neuregulin stimulation with release of prostaglandin E_2_, a key mediator used by astroglial cells of the neuroendocrine brain to facilitate female reproductive development.

During the course of these experiments, we noticed that GFAP-DNSynCAM1 mice exhibited an unusually high level of activity, which appeared unabated during the light period of the light cycle, suggesting that their diurnal pattern of locomotor and/or sleep activity was compromised. The mutant animals also appeared to display a persistent, but aimless pattern of exploratory behavior in a familiar environment. In addition, they exhibited increased impulsivity as evidenced by a tendency to jump from the cage when the lid was removed, and to attack other animals or the person opening the cage without provocation. To characterize some of these alterations we subjected the animals to a battery of behavioral tests measuring diurnal patterns of locomotor activity, anxiety, motor coordination, and response to amphetamine administration. The results of these analyses revealed that GFAP-DNSynCAM1 mice display behavioral manifestations previously observed in mouse models of attention deficit hyperactive disorder (ADHD) [15], [16]. Because GFAP-DNSynCAM1 animals have an astrocyte-specific defect in SynCAM1 signaling, alterations in astrocyte function requiring adhesive-dependent cell-cell communication might contribute to the neurodevelopmental defects underlying the behavioral consequences of ADHD.

## Materials and Methods

### Animals

Male heterozygous mice that express an astrocyte-specific dominant-negative form of SynCAM1 (GFAP-DNSynCAM1) under control of the glial fibrillary acidic protein (GFAP) promoter on the FvB/N background [14] were bred to either FvB/N or C57BL/6 J wild-type (WT) females. Three independent transgenic lines of GFAP-DNSynCAM1 mice (Lines 27, 42 and 45) were used to generate offspring. Heterozygous adult male littermates from Lines 27 (n = 8) and 45 (n = 6) and WT littermates (n = 8) were used to study changes in diurnal locomotor activity. The animals employed were first generation (F1) adult male mice on an outcrossed FvB/N×C57BL/6 J background. We used these F1 mice, because the FVB/N strain is homozygous for the retinal degeneration 1 allele (*Pde6b^rd1^*), which results in early onset retinal degeneration. The F1 progeny of this cross would be expected to recover normal retinal function while maintaining genetic homogeneity between experimental mice. Although we observed that mice maintained on the FvB/N background had a pattern of locomotor activity similar to that of the F1 animals (data not shown), we used F1 animals for all experiments assessing diurnal changes in locomotor activity. The F1 mice were weaned at 21 days of age and housed with littermates of both genotypes until they were 4–5 months old. Thereafter, they were separated into individual housing 3 days prior to implantation surgery, and remained individually housed until completion of the study. Line 42 adult male mice (n = 8) and WT littermates (n = 9) maintained on the original FvB/N background were used for all other behavior tests. We employed L42 animals for these tests because in our initial characterization of the mutants we found that the GFAP-DNSynCAM1 transgene was more abundantly expressed in areas of the brain more relevant to behaviors of interest than the hypothalamus. Thus, L42 mice were expected to be a better model for behavioral alterations than L45 and 27 in which the DN-SyCAM1 transgene is more abundantly expressed in the hypothalamus than the rest of the brain. L42 male pups were weaned at 21 days of age and housed with littermates of the same genotype until 4–5 months of age. Due to the aggressive tendencies of the GFAP-DNSynCAM1 mice, WT males were kept separate from mutant males to avoid potential fear conditioning prior to testing. All animals were single housed throughout the duration of the tests. Genotyping was carried out using PCR protocols previously described [14]. The animals were housed under a 12∶12 h light - dark cycle (lights on at 0600) and given free access to food and water. All experiments were conducted in accordance with NIH guidelines and approved by the Oregon National Primate Research Center/Oregon Health & Science University Institutional Animal Care and Use Committee.

### Behavioral Tests

Adult male mice were tested for (i) diurnal activity using MiniMitter implantation devices (MiniMitter, Sun River, Oregon); (ii) spontaneous locomotor activity and altered amphetamine response using the open field test and infrared photo beams (Kinder Scientific, Poway, California); (iii) measures of anxiety involving avoidable anxiety-provoking stimuli using the elevated zero maze test (Kinder Scientific) and (iv) measures of anxiety involving unavoidable anxiety-provoking stimuli induced by acoustic stimuli in sound-attenuated acoustic startle chambers (Kinder Scientific). DN-SynCAM1 mice were also tested for deficits in sensorimotor function on a rotarod, and learning and memory using contextual and cued fear conditioning tests, as described below in detail.

Tests ii-iii were conducted in the following order: Week 1 – elevated zero maze, rotarod, acoustic startle, and pre-pulse inhibition; Week 2 – cued and contextual fear conditioning; Week 3 - no behavior testing, animals remained in the home cage, but were handled daily by the investigator; Week 4 - spontaneous locomotor activity and response to amphetamine.

### Mini Mitter

To assess changes in diurnal activity in the home cage, mice were implanted under isoflurane anesthesia with pre-calibrated sensitive transmitters (PDT-4000 G2 E-Mitter® sensors, Mini Mitter Company, Sun River, OR). E-Mitters were implanted beneath the interscapular brown adipose tissue adjacent to the right scapula. The devices were secured to muscle tissue with a single suture, and wounds were closed with MikRon stainless steel wound clips. For the actual experiment, the mice were moved from the colony room to a separate isolated study room. Both rooms shared the same 12-hour light cycle. Human activity in the room was limited to necessary husbandry and technician duties. Mice were allowed 1 week of recovery and acclimation to the new environment before studies commenced. Mouse activity was then recorded for 1 week. Cages were not changed during these 2 weeks so that the mice would not be disturbed. Signals emitted by the E-Mitter transmitters were detected by a receiver positioned underneath the animal's home cage and converted into activity counts (arbitrary units) by VitalView® software (Mini Mitter). Locomotor activity counts measured this way are a relative measure of gross motor activity. For all experiments, activity counts and interscapular temperature measurements were recorded every 6 minutes. Activity count data was calculated as cumulative activity per 6-minute interval. Temperature data was also recorded and calculated by the VitalView® software using a weighed time-averaged “bin” based on sample data points taken at 6-minute intervals. All temperature data points more than one standard deviation away from the mean were discarded by the software. Once data collection was complete, the raw data was exported from VitalView® in the form of a delimited ASCII file and imported into a Microsoft Excel spreadsheet for further analysis.

### Open Field

*S*pontaneous locomotor activity and response to amphetamine was assessed in the open field arena over three periods: habituation, saline injection and amphetamine injection. The open field arena (16 inch×16 inch square) was equipped with a 16×16 array of infrared photocells for measuring horizontal movements and computer-quantified automatically (Kinder Scientific, Poway CA). The open field test was conducted during the active (lights out) period of the animal's 12∶12 cycle, starting at the time of lights off. Animals were placed into the open field arena for 4 consecutive hours for habituation. After habituation, the animals received a subcutaneous saline injection (volume matching that of the subsequent amphetamine injection) and returned to the open field for 2 consecutive hours to assess locomotor activity in response to a vehicle injection. After the saline trial, animals received a subcutaneous injection of D,L- amphetamine (4 mg/kg) and were returned to the open field to assess their response to amphetamine. The animals were tested in their own dedicated chambers for the duration of the night and were not disturbed during testing periods. After each trial, the open field arena was cleaned with 0.5% acetic acid. During this time, the animals were returned to their home cage for no more than a 5-minute period. The total distance traveled by each animal during each period was analyzed to determine the activity levels.

### Elevated Zero Maze

Measures of anxiety involving avoidable anxiety-provoking stimuli were assessed using the elevated zero maze. The elevated zero maze consisted of two enclosed areas (safe environments) and two open areas (anxiety-provoking environment), identical in length (35.5 cm; Kinder Scientific, Poway, CA). The elevated zero maze was equipped with infrared photocells for measuring horizontal movements and computer-quantified automatically (Kinder Scientific, Poway CA). Mice were placed into the maze facing the enclosed area at a site where the enclosed and open areas interfaced. The mice were allowed free access to the maze for 5 min. The software calculated the total distance moved; distance moved in both the enclosed and open areas; and time spent active and resting in the enclosed and open areas of the maze.

### Acoustic Startle

Measures of anxiety involving unavoidable acoustic stimuli were assessed using the acoustic startle behavioral paradigm. Animals were placed in a plexiglass enclosure on a sensing platform within a sound-attenuating chamber (Kinder Scientific, Poway, CA). After a 5-min acclimation, the baseline response was measured in three trials. Animals were then exposed to pulses of white noise administered in increments of 2 db, from 80–120 db within a 500 msec window, and the maximum force of the mouse on the sensing plate was measured in newtons (N). Startle amplitude was defined as the peak voltage force that occurred during the 500 msec record window. Wideback background noise (72 db) was used during testing. For each trial, the startle amplitude was computer-quantified automatically using Kinder Scientific software (Poway, CA).

### Rotarod

Sensorimotor performance was assessed on a Rotarod. Mice were placed on an elevated rotating rod (diameter: 3 cm, elevated: 45 cm, Rotamex-5, Columbus Instruments, Columbus, OH), initially rotating at 5.0 RPM. The rod accelerated 1.0 RPM every 3 seconds. A line of photobeams beneath the rod recorded the latency to fall(s). Each mouse received three trials per day, with no delay between trials, on three consecutive days.

### Fear Conditioning

The mice were tested for fear conditioning using Med Associates NIR Video and automated analysis (Med Associates, St. Albans, Vermont) utilizing Med Associates Video Freeze automated scoring system. In this task, mice learn to associate a conditioned stimulus (CS, e.g. a tone) with a mild foot shock (unconditioned stimulus, US). CS-US pairings are preceded by a short habituation period, from which a baseline measure of locomotor activity and other behavior can be scored. Post-exposure freezing, defined as absence of all movement with the exception of respiration, is a widely used indictor of a conditioned fear response. On day 1, the mice were placed inside a white LED lit (100 lux) fear conditioning chamber (Context A). There was a 150 second baseline followed by two CS-US pairings. A 2.8 kHz, 80 dB tone (CS) was presented at 150 and 270 seconds. Both CS presentations co-terminated with a 2-sec 0.35 mA footshock (US). On day 2, hippocampus dependent associative learning was assessed during re-exposure to Context A for 300 seconds. Three hours later, mice were exposed to Context B. Context B contained a smooth white floor, a black plastic triangular insert for the walls, scented with vanilla. The mice were allowed to habituate for 180 seconds (Pre-CS period), and then exposed to the tone for a second period of 180 seconds (Post-CS period). Associative learning was measured as the percent time spent freezing in response to the contextual environment or tone. Motion during shock (arbitrary units) was measured to explore potential genotype differences in response to the aversive stimulus.

### Pre-pulse inhibition (PPI)

Mice were placed in an enclosure within a startle monitor sound-attenuated chamber and startle response amplitudes were measured with a force transducer (Kinder Scientific, Poway, CA). Following a 5 min acclimation period, mice were exposed to 3, 40 ms acoustic stimuli (110 db). The testing phase consisted of 20 ms pre-pulses (70–80 db) followed by 50 ms delays and 40 ms acoustic stimuli (110 db). The same pattern of acoustic stimuli and testing with pre-pulses then occurred with a 120 db stimulus. Random inter-trial intervals were used between trials (15–30 sec). PPI was calculated using the following formula: % response = 100×(S-PS)/S), where S was the mean startle amplitude without a pre-pulse and PS was the mean startle response following a pre-pulse. Thus, a 100% response in the PPI test indicates complete inhibition of the startle response during pre-pulse trials.

### Statistical Analyses

All statistical analyses were performed using Statview software version 5.0.1 (SAS Institute Inc, Cary, NC) or GraphPad Prism software (GraphPad Software, La Jolla, CA). Two way ANOVAs were used for analysis of the diurnal activity data using genotype as between-subject factor and light/dark cycle as within-subject factor. To analyze the open field data and acoustic startle data, repeated measures ANOVAs were performed using genotype as a between-subject factor and time post injection or stimulus intensity (db) as the within-subject factor. Depending on the outcome of the ANOVA analysis, post-hoc tests were used when appropriate. T-test was used for analysis of the elevated zero maze data. Data were expressed as means ± SEM. p<0.05 was considered significant for all tests.

## Results

### GFAP-DNSynCAM1 mice show signs of increased impulsivity

Throughout the duration of the study, we observed (but did not quantify) behavioral features in GFAP-DNSynCAM1 mice suggestive of increased impulsivity. They had a tendency to jump from the cage upon removal of the cage's lid. They also appeared more aggressive as they attacked other mice without provocation and persistently attempted to bite the hand of investigators and animal technicians attempting to handle them. Because of this, it was necessary to always wear a Kevlar glove when handling them.

### GFAP-DNSynCAM1 mice display increased home cage activity and attenuation of changes in diurnal rhythm activity

**Figure 1:**
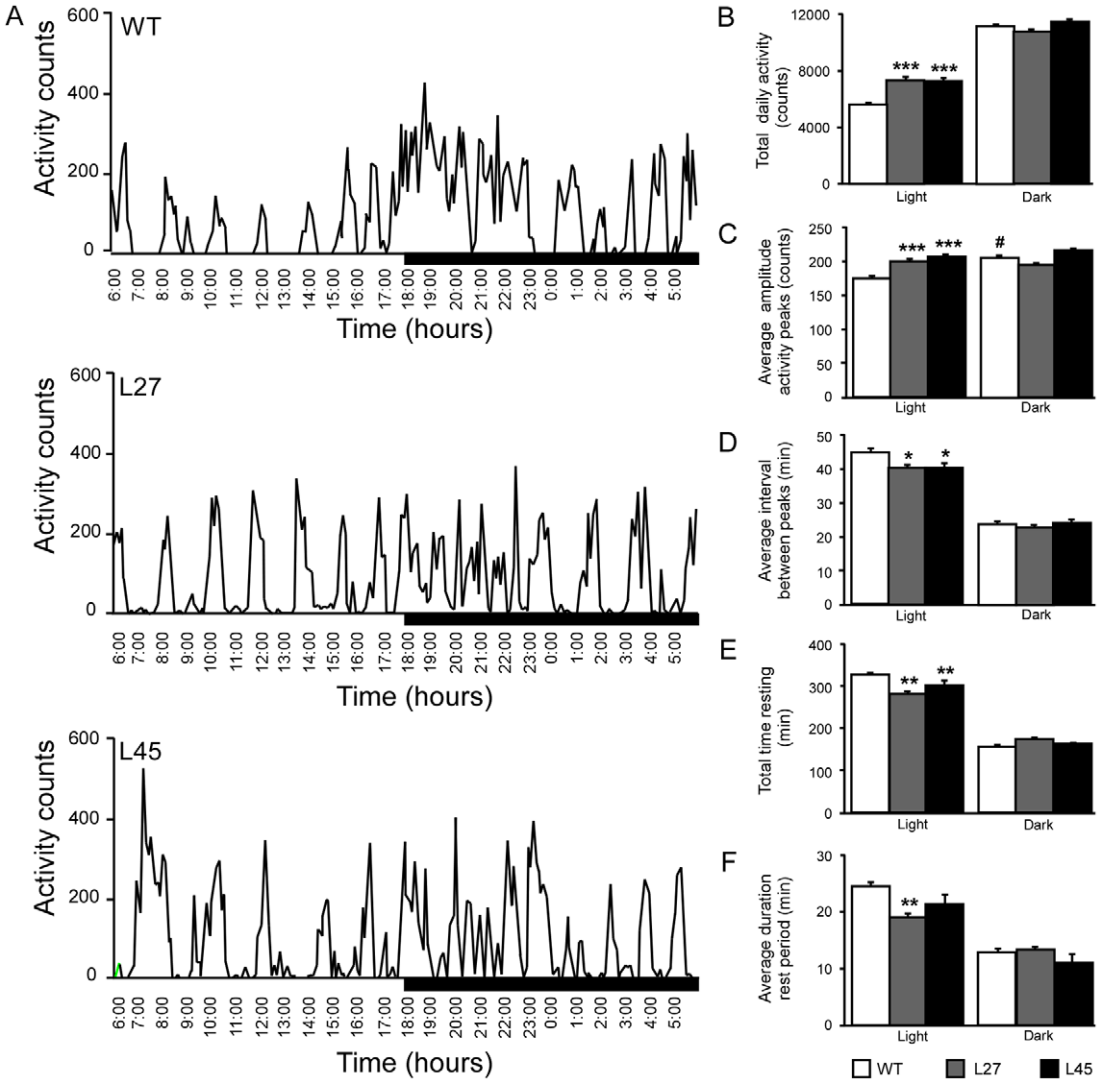
GFAP-DNSynCAM1 mice have increased locomotor activity and shortened resting intervals during a 24 h period. Adult male GFAP-DNSynCAM1 L27 (n = 8), L45 (n = 6) mice and WT littermates (n = 8) were housed individually and 24-hour activity was recorded in 6-minute bins for a period of one week using MiniMitter telemetry devices. (**A**) Representative 24-hour activity profiles from one WT littermate (upper panel) and two GFAP-DNSynCAM1 adult male mice (Line 27, middle panel; L45, lower panel). (**B**) Average cumulative daily activity counts for both light and dark periods. (**C**) Average amplitude of activity spikes during light and dark periods (number of activity counts per 6-minute bin). (**D**) Average frequency of activity peaks (minutes) for light and dark periods. (**E**) Average cumulative time spent resting (minutes) during light and dark periods (6-minute bins recorded as zero movement). (**F**) Average duration of each rest period (minutes) for both light and dark periods. Activity was analyzed in the light and dark periods for WT (white bars), DN-SynCAM1 L27 (grey bars) and DN-SynCAM1 L45 (black bars). For all graphs data are expressed as the mean ± SEM. Data are analyzed using a two way ANOVA (period by genotype) followed by Student-Newman-Keuls post hoc analysis, * p<0.05; ** p<0.01; *** p<0.001.

**Figure 2:**
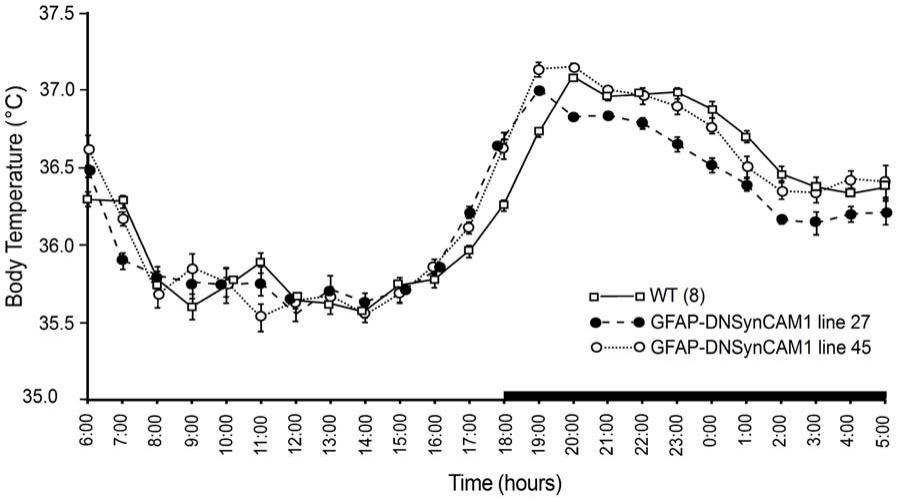
Average 24-hour body temperature profiles. F1 adult male GFAP-DNSynCAM1 L27 (n = 8), L45 (n = 6) mice and WT littermates (n = 8) of an FvB/N×C57BL/6 J background were housed individually and 24-hour body temperature was recorded in 6-minute weighted time-averaged bins for a period of one week using MiniMitter telemetry devices. The mutant mice have a 1-hour phase shift relative to WT littermates in the peak of body temperature increase that occurs as the animals enter the dark phase.

Twenty four-hour home cage activity of two independent lines of GFAP-DNSynCAM1 (L27 and L45) mice and WT littermate controls was recorded using implanted MiniMitter devices for a period of one week. WT mice exhibited a diurnal rhythm of locomotor activity characterized by a decrease in spontaneous activity with a corresponding increase in the duration of resting periods (the interval between activity peaks) during the light phase as compared to the dark phase (**Fig. 1A**, upper panel). The activity profiles of GFAP-DNSynCAM1 mice were strikingly different, showing more pronounced episodes of activity during the light phase than WT controls (**Fig. 1A**, middle and lower panels). The mutant mice had a significant increase in total daily activity during the light phase compared to WT littermates (*F = 17.04; p<0.0001*) (**Fig. 1B**). They also exhibited a significant (*F = 36.33; p<0.0001*) increase in the amplitude of activity peaks during this phase of the light cycle (**Fig. 1C**). In contrast to WT mice, in which the peak amplitude of each activity episode was significantly (p<0.0001) higher at night than during the day, GFAP-DNSynCAM1 mice showed no differences in peak amplitude between the light and dark phase of the light cycle (**Fig. 1C**). Episodes of activity during the light phase occurred more frequently (*F = 4.45; p = 0.019*) in the mutant mice than in WT animals (**Fig. 1** **D**). The transgenic mice also spent significantly (*F = 3.35; p<0.047*) less total time resting than WT animals during the light phase (**Fig. 1E**), and their rest periods were of a shorter (*F = 3.82; p<0.031*) duration (**Fig. 1F**). Average hourly body temperature showed a diurnal pattern that was similar between GFAP-DNSynCAM1 mice and WT controls. In both cases, body temperature began to increase by 16:00 h, andreached peak levels within the first two hours of the dark phase (**Fig. 2**). The peak occurred one hour sooner in the mutant mice than in WT littermates (**Fig. 2**).

### GFAP-DNSynCAM1 mice exhibit increased spontaneous locomotor activity in the open field that is attenuated by amphetamine

**Figure 3:**
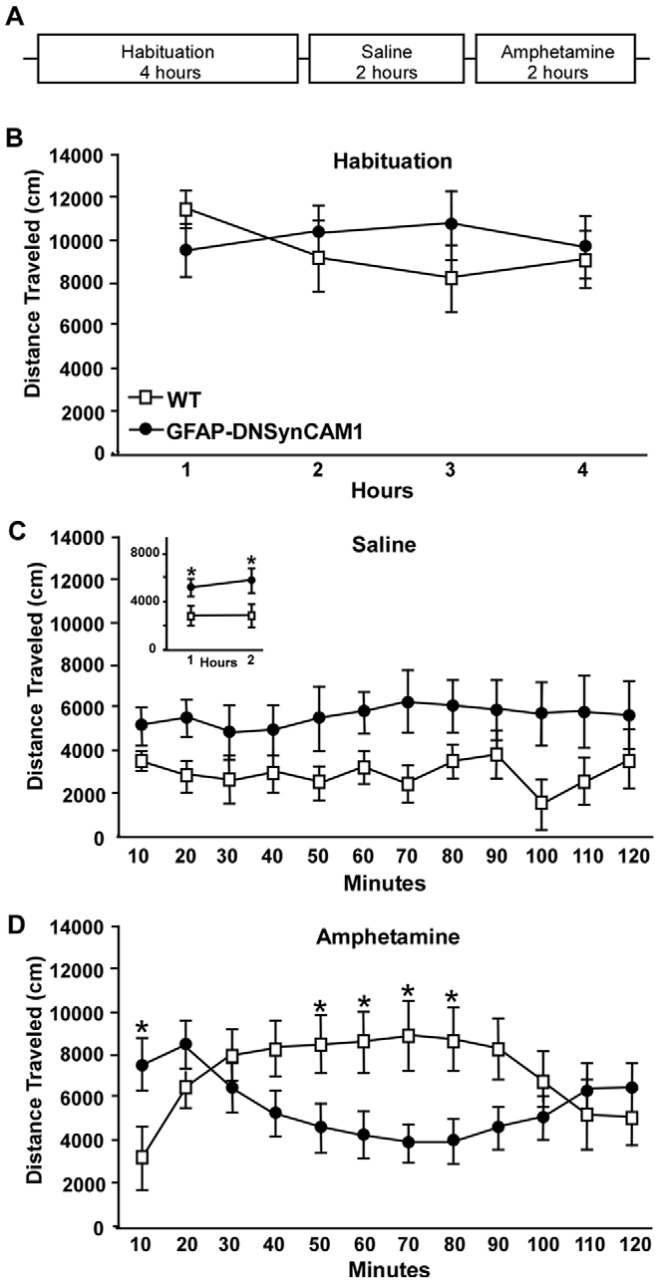
GFAP-DNSynCAM1 mice have increased spontaneous locomotor activity that is attenuated by amphetamine. (**A**) Illustration depicting the behavior paradigm used to assess spontaneous locomotor activity and response to amphetamine in the open field. Adult male GFAP-DNSynCAM1 (n = 8) and WT littermates (n = 5) were subjected to the following paradigm during the dark (active) phase of the light cycle: 4 h of habituation, followed by 2 h of spontaneous locomotor activity post saline injection, followed by 2 h of locomotor activity after injection of D,L- amphetamine (4 mg/kg). (**B**) Total distance traveled (cm) in 1 hour time bins by GFAP-DNSynCAM1 (black circles) and WT littermates (white squares) during the habituation period. (**C**) Total distance traveled (cm) in 10 minute time bins by GFAP-DNSynCAM1 (black circles) and WT littermates (white squares) immediately following a subcutaneous injection of saline. Inset is the average distance traveled (cm) by each mouse during the first and second hours of behavior assessment. (**D**) Total distance traveled (cm) in 10 min bins by GFAP-DNSynCAM1 (black circles) and WT littermates (white squares) immediately following a subcutaneous injection of D,L- amphetamine (4 mg/kg). For all graphs the data points are represented as means ± SEM. Panels B–D were analyzed using a repeated measures ANOVA (time by genotype) followed by an unpaired t-test for each time point (panel D). Data shown in the inset to panel C were analyzed by a two way ANOVA (time by genotype) followed by a Student-Newman-Keuls post hoc test. * p<0.05.

Spontaneous locomotor activity of GFAP-DNSynCAM1 mice was tested during the active (dark) phase of the animal's cycle as illustrated in **Fig. 3A**. During the 4-h habituation period the mutant and WT littermates had similar activity levels. However, upon injecting the animals with saline and returning them to the open field (Saline 2 h), the transgenic mice showed an increase in locomotor activity (**Fig. 3B, C**). This increase was sustained throughout the 2 h period studied (**Fig. 3C**), with values significantly higher (*F = 7.66; p = 0.011*) in GFAP-DNSynCAM1 as compared to WT mice during both the first and the second hour following saline injection (**Fig. 3B** **, inset**). The increase in activity shown by GFAP-DNSynCAM1 mice occurred independent of significant changes in sensorimotor function (measured using an accelerating rotarod), potential deficits in learning and memory (assessed using contextual and cued fear conditioning paradigms) or alterations in acoustic startle or pre-pulse inhibition (data not shown).

To determine if the increased locomotor activity of GFAP-DNSynCAM1 mice was associated with an altered response to amphetamine, mice were administered a single subcutaneous injection of the drug (4 mg/kg) and immediately returned to the familiar open field arena. Across the 2 h testing period there was a highly significant genotype by time bin effect (*F = 5.88; p<0.0001*; **Fig. 3D**). In the first 10 min time bin the GFAP-DNSynCAM1 mice had a significant (*p<0.025*) increase in total distance traveled compared to the WT littermates, which is in accord with the baseline activity measurements shown in **Fig. 3B**. However, by 30 minutes after amphetamine the GFAP-DNSynCAM1 mice began to respond to amphetamine with a decrease in total distance traveled; in contrast, WT littermates responded to amphetamine with increased locomotor activity (**Fig. 3D**). This altered response to amphetamine in the transgenic mice was significantly (*p<0.05*) different from the response of WT littermates between 50 and 80 minutes post injection. After this interval, activity levels in both genotypes began to return to baseline.

### GFAP-DNSynCAM1 mice have decreased measures of anxiety

**Figure 4:**
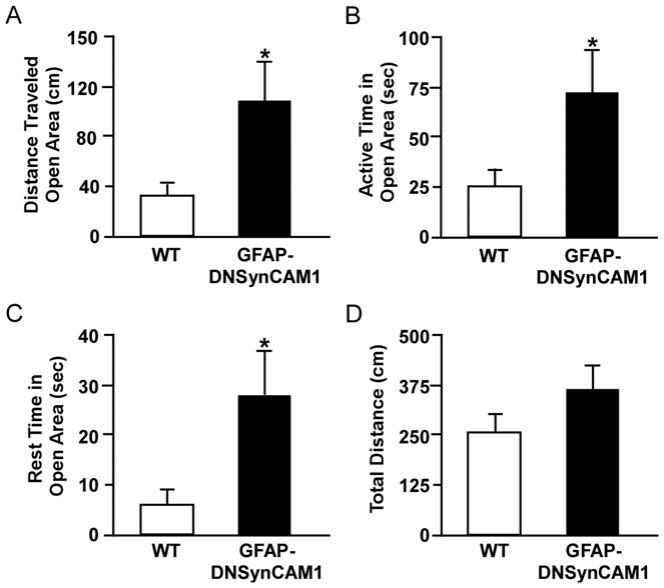
DN-SynCAM1 mice display behaviors of reduced anxiety in the zero maze. (**A**) Total distance traveled (cm) by GFAP-DNSynCAM1 (black bars; n = 9) and WT littermates (white bars, n = 8) in the open areas of the zero maze. (**B**) Total active time (seconds) in the open areas for GFAP-DNSynCAM1 (black bars) and WT littermates (white bars). (**C**) Total rest time (seconds) in the open areas for GFAP-DNSynCAM1 (black bars) and WT littermates (white bars). (**D**) Total distance traveled in the open and closed areas of the zero maze by GFAP-DNSynCAM1 (black bars) and WT littermates (white bars). Data are represented as the mean ± SEM and compared using an unpaired t-test. * p<0.05.

In addition to an increase in spontaneous locomotor activity and altered response to amphetamine, transgenic mouse models of ADHD have either reduced or unchanged measures of anxiety [17], [18]. Thus, we sought to determine if GFAP-DNSynCAM1 mice exhibit changes in measures of anxiety using paradigms involving avoidable and unavoidable anxiety-provoking stimuli. Transgenic and WT type littermates were first tested in the elevated zero maze (**Fig. 4**). GFAP-DNSynCAM1 showed reduced measures of anxiety. They travelled a longer distance (*p = 0.031*; **Fig. 4A**) and had increased active (*p = 0.035*) and rest times (*p = 0.045*) within the open areas, as compared to the WT controls (**Fig. 4B and C**). Conversely, the total distance traveled in both the open and closed areas was similar in the transgenic and WT littermates (**Fig. 4D**).

**Figure 5:**
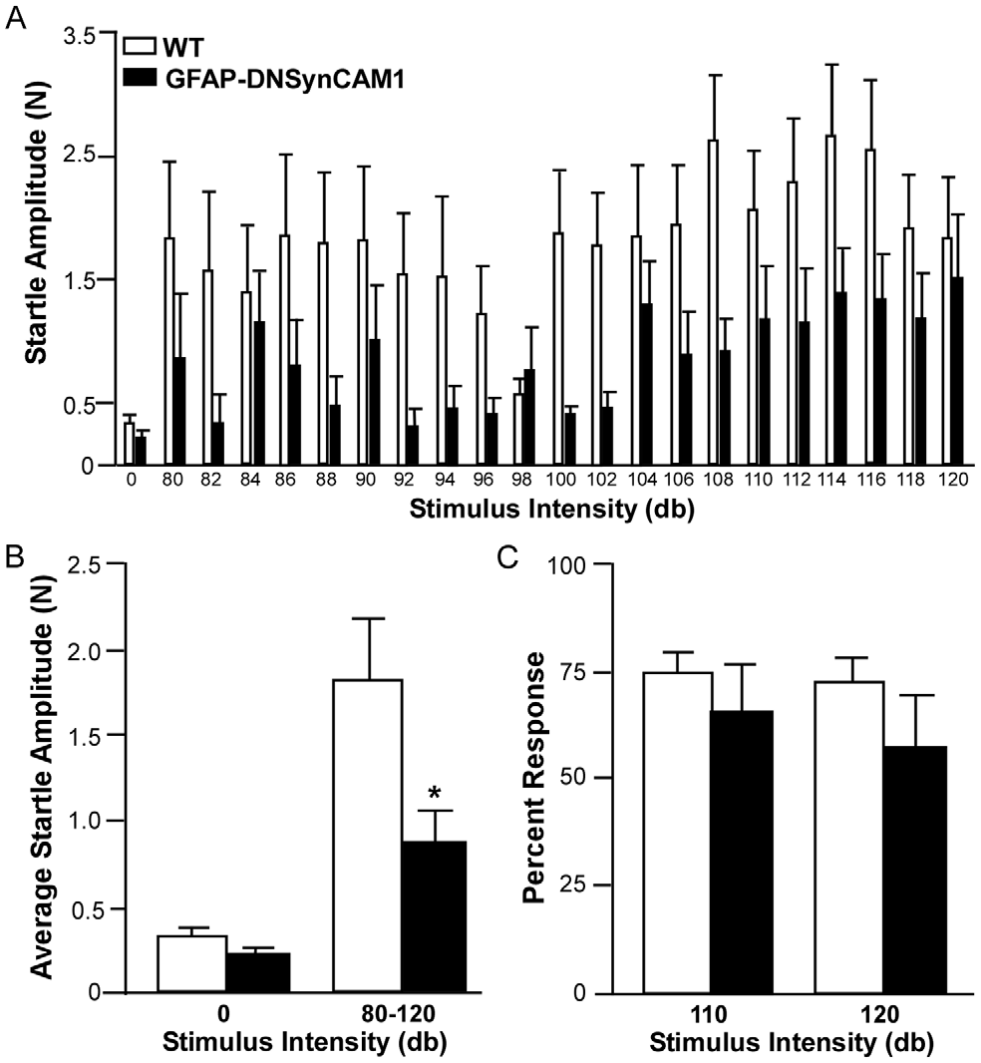
GFAP-DNSynCAM1 mice have reduced anxiety in the acoustic startle paradigm. (**A**) Startle amplitudes for the GFAP-DNSynCAM1 (black bars; n = 8) and WT (white bars; n = 7) mice for each of the sound intensities tested. Data points represent means ± SEM. Data were analyzed with ANOVA for repeated measures. (**B**) The average startle amplitudes for each genotype during baseline (0 db) and after different stimulation intensities (80–120 db). (**C**) Percent response of GFAP-DNSynCAM1 (black bars; n = 9) and WT (white bars; n = 8) in the PPI test using two different stimuli intensities (110 and 120 db). The data are represented as the mean ± SEM and analyzed by two- way ANOVA (intensity by genotype) followed by unpaired t test post hoc analysis. * p<0.05.

The GFAP-DNSynCAM1 mice also displayed reduced measures of anxiety in the acoustic startle test. Across all stimulus intensities tested there was a significant genotype effect (*F = 6.33; p = 0.0258*) showing a lower startle amplitude in the GFAP-DNSynCAM1 mice compared to the WT controls (**Fig. 5A**). Subsequent analysis of the average startle amplitudes for the WT and DN-SynCAM1 mice at baseline (0 db) versus all other stimulation intensities (80–120 db) indicated a genotype×stimulation condition interaction (*F = 26.16; p = 0.0002*; **Fig. 5B**). While there was a significant reduction (*p<0.05*) in the average startle amplitude in the GFAP-DNSynCAM1 mice within the 80–120 bd range compared to WT controls, there was no difference in the two groups at 0 db. Finally, we assessed the GFAP-DNSynCAM1 mice for deficits in sensorimotor gating using the PPI test. The percent response to an acoustic startle (110 or 120 db) following a pre-pulse was not significantly different in the GFAP-DNSynCAM1 mice compared to the WT controls (**Fig. 5C**).

## Discussion

The present results demonstrate that an alteration of glial biology caused by loss of SynCAM1-dependent signaling specifically targeted to astrocytes results in behavioral manifestations previously observed in rodent models of ADHD. The increased impulsivity, disruption of the light-dark patterns of locomotor activity, and the paradoxical calming effect of the pysochostimulant amphetamine on hyperactivity are all hallmarks of ADHD [15], [16], [19], and are all observed in mutant mice with disrupted astroglial SynCAM1 function.

ADHD is a highly heritable condition that affects 6–9% of children and that is characterized by three main behavioral abnormalities: hyperactivity, impulsivity and impaired sustained attention [15], [19]. Although ADHD is considered a neurodevelopmental disorder of childhood [20], its manifestations persist well into adulthood [19]. The present study, carried out in adult male mice, is consistent with this concept. The precise etiology of ADHD remains elusive, but genetic analyses of affected human populations have shown association of ADHD with mutations in several genes involved in synaptic transmission, and specifically with genes encoding components of the monoaminergic neurotransmitter system. Prominent among these genes are *SNAP25*, which encodes a presynaptic protein required for neurotransmitter release [21], [22], *DRD2*, the gene encoding dopamine (DA) receptors of the 2 subtype [23], and *DAT1* [22], [24], [25], which encodes a dopamine transporter responsible for clearing DA released into the synaptic cleft during synaptic activity. The serotonin receptor 5HT1b and serotonin transporters have also been implicated [17], [22], [26].

The genetics of ADHD [22], [24], [26], and results obtained using mouse models of the disorder [15], [16], support the concept that impairments in synaptic neurotransmission are a major underlying cause of most ADHD manifestations. Nevertheless, none of the available mouse models have provided data free of inconsistencies, suggesting the existence of additional mechanisms independent of a primarily neuronal impairment in synaptic transmission. For instance, the high blood pressure observed in spontaneous hypertensive rats (SHR), the most widely used animal model of ADHD, is not a feature of ADHD. When hypertension is eliminated as a confounding factor by crossing SHR rats to WKY rats, the resulting WKHA rat has normal blood pressure, but no longer responds to the psychostimulant methylphenidate with decreased hyperactivity [27]. The Coloboma mouse, another frequently used mouse model of ADHD, has mutations in the genes encoding SNAP-25 and phospholipase C-β1 [15], [16], and exhibit profound hyperactivity, which is reduced by amphetamine, but not by methylphenidate [21]. Mice lacking the DA transporter DAT1 respond to both amphetamine and methylphenidate with decreased hyperactivity, but surprisingly this effect occurs even when these animals lack DAT, a protein shown by other studies to be a major target for both stimulants [15], [16].

Thus, additional molecules and other mechanisms may contribute to the physiopathology of ADHD. Without negating the importance of pure impairments in neurotransmission, our results suggest that the astrocyte-specific loss of SynCAM1-mediated signaling is a contributing factor. Neurons may not be directly affected in GFAP-DNSynCAM1 mice, because mutations in *CADM1* (the SynCAM1 gene in humans) that cause morphological abnormalities in neurons and defects in synaptogenesis, are not associated to ADHD, but instead appear to contribute to the molecular pathogenesis of autism spectrum disorder [28]. This is an important consideration because the construct we employed to target mutant SynCAM1 to astrocytes is driven by the GFAP promoter, and GFAP has been shown to be expressed in neural progenitor cells of the adult mouse forebrain [29]. In fact, GFAP-expressing progenitor cells have been shown to be responsible for the bulk of constitutive neurogenesis in the adult brain [29]. However, in other studies using transgenes driven by the same GFAP promoter we did not observe changes in a variety of basic physiological parameters of neuronal function [30]. Another cell type expressing a polysialylated form of SynCAM1 is the NG2 cells [31], which are scattered throughout the brain and give rise to oligodendrocytes, astrocytes, and neurons. Since these cells do not express GFAP, it unlikely that they may have been directly affected in the GFAP-DNSynCAM1 mice. Thus, the effects we observe appear to be specific to alterations in astrocytic SynCAM1 function.

In addition to ADHD-like impairments, GFAP-DNSynCAM1 mice exhibit reduced measures of anxiety, and increased impulsivity, two features observed in serotonin receptor 5HT_1B_ KO mice [17], another model of ADHD. Although co-morbid anxiety conditions are common in children with ADHD [19], they do not constitute an integral feature of the disorder. Instead, animal studies suggest that measures of anxiety are either reduced [17] or unchanged [18] in ADHD.

The disruption in diurnal rhythms of activity observed in GFAP-DNSynCAM1 mice is characterized by more frequent and intense episodes of activity during the day – when the animals are normally sleeping – accompanied by more infrequent and shorter periods of rest. Although we did not evaluate sleep architecture directly, this disrupted pattern of diurnal activity strongly suggests that GFAP-DNSynCAM1 mice might also suffer from sleep disturbances, another hallmark of ADHD [19]. An involvement of astroglial cells in sleep regulation is now well established. Astrocytes control sleep homeostasis by releasing ATP, which is cleaved into adenosine [32] a purine nucleoside that promotes sleep [33]. Astrocytes play a further role in regulating extracellular adenosine concentrations through passive reuptake of adenosine followed by adenosine kinase-mediated intracellular phosphorylation of adenosine to adenosine-5′-monophosphate (AMP) [34]. This regulatory effect is likely to have a diurnal periodicity because ATP is released from astrocytes in a circadian fashion [35]. In humans, sleep disturbance associated with ADHD have been ascribed to polymorphism of the DNA sequence encoding the 3′ untranslated region of the *CLOCK* gene [36], [37], a gene essential for circadian periodicity [38], [39].

The ADHD-like manifestations of GFAP-DNSynCAM1 mice may be due to an inability of astrocytes to communicate with other astrocytes and neurons via adhesive-mediated signaling. Like many other adhesion molecules of the immunoglobulin superfamily [2], SynCAM1 is capable of signal transduction. Its intracellular domain contains both a FERM binding motif and a C-terminus sequence recognized by proteins containing PDZ domains type II [3], [40]. Binding of membrane associated proteins (such as protein 4.1, calcium/calmodulin-dependent serine protein kinase [CASK] and syntenin) to these domains, link SynCAM1 to signaling events associated with the structural re-organization of the cytoskeleton [40], [41]. The FERM domain also recruits NMDA and AMPA receptors for excitatory neurotransmission [42]. Because astrocytes express both metabotropic glutamate and AMPA receptors, which upon activation result in erbB receptor-mediated glia-to-neuron signaling events [43], [44], it is possible that a defect in astroglial SynCAM1 function results in ADHD-like behaviors because of an alteration in excitatory glia-to-neuron communication. This notion is supported by the finding that *Grin1* mutant mice, which carry a mutation of the NMDA receptor subunit 1, exhibit ADHD-like enhanced locomotor activity that is methyphenidate-sensitive [18]. Alterations of glutamatergic transmission in ADHD are further suggested by the reduction in glutamate release observed in cortical synaptosomes from Coloboma hyperactive mice [45]. A role for astrocytes in this process is implied by the well- established contribution of astrocytes to the regulation of excitatory neurotransmission [46], [47].

We earlier observed that SynCAM molecules cluster in both astrocytes and neurons as a result of erbB4 receptor activation [14], leading to the formation of “hot-spots” of cell adhesion and signaling domains. These domains may contribute to the dynamics of receptor distribution that occurs in both neurons and astrocytes in response to changes in cell activity. Their formation also appear to require CASK, because CASK is recruited by SynCAM1 to the cell membrane [6], promotes cytoskeleton reorganization [48], sorts NMDA receptors [49], and interacts with N- and P/Q-type of voltage-gated calcium channels [50], [51]. Thus, SynCAM1 may act on astrocytes not only to regulate cell plasticity, but also to facilitate calcium influx and release of excitatory neurotransmitters.

Although astrocytic SynCAM1 is functionally linked to erbB4 receptor function, and alterations in ligand-induced erbB4 signaling have been associated to the physiopathology of schizophrenia [[52], [53] and references therein], we did not observe differences in PPI between GFAP-DNSynCAM1 mice and their WT littermates. PPI of the acoustic startle response, a measure of sensorimotor gating, is widely used in rodent models of schizophrenia, because it is consistently diminished in schizophrenic patients [54]. Since we measured PPI only under basal conditions, we cannot exclude the possibility that a schizophrenia-like behavior would have been unveiled in DN-SynCAM1 mice treated with amphetamine [55], [56].

Altogether, our findings support the concept that SynCAM1-dependent deficiency in astrocytic function contribute to the physiopathology of ADHD. They also suggest that the behavioral manifestations caused by these deficiencies result from an inability of astrocytes to establish functional avenues of cell-cell communication with relevant neuronal networks. Future studies are warranted to determine the age at which these behavioral impairments are initiated, the cellular mechanisms involved, and whether increasing astrocytic SynCAM1 levels can reduce or even prevent ADHD-like behaviors.

After our manuscript was accepted we became aware of a paper by Shamir A et al. (J.Neuroscience 32:2988–2997, 2012) reporting very similar behavioral phenotypes in mice lacking the ErbB4 receptor.
